# The Hospital Nursing Supplement

**Published:** 1893-09-02

**Authors:** 


### "The Hospital" Nursing Mirror.

Being the Extra Nursing Supplement of "The Hospital" Newspaper.

[Contributions for this Supplement should be addressed to the Editor, The Hospital, 428, Strand, London, W.C., and should have the word "Nursing" plainly written in left-hand top corner of the envelope.]

### News from the Nursing World.

### OUR CHRISTMAS COMPETITIONS.

Many readers are just completing, or have already completed their holidays, and so are able, with restored health and energy, to turn their attention to the daily round. It is most desirable that a much larger number than ever before should enter for this competition. We anticipate that an immense variety of garments will this year be made by our readers for the adult-sick poor who have to spend their Christmas Day within hospital walls. We find that the parcels which are the result of our annual competition receive such hearty welcomes at the institutions to which they are sent, that we hope to see them increase in bulk and in number each year. The prizes will be: (1) For the best pair of socks knitted by a nurse, 5s.; (2) for the best pair knitted by any Hospital reader, 5s.; (3) for the best made flannel shirt, 10s.; (4) for the best flannel or flannelette bed-jacket, 10s.; (5) for the best flannel petticoat, 10s.; (6) for the best made and simplest shaped dressing gown made and cut out by a nurse, 20s. Long seams may be done by machine. With the view of enabling an estimate to be formed of the progress made, we shall be glad to receive the names of competitors, and to hear which of the six prizes each will enter for.

### THE NIGHTINGALE FUND.

The thirty-second report of the Nightingale Fund is signed by Sir Harry Verney, chairman, and Mr. H. Bonham-Carter, the secretary, two veterans who have earned the gratitude and admiration of all who take an intelligent interest in trained nurses. We are glad to notice that due recognition is given to the excellence of the arrangements of Miss Gordon and Miss Crossland, and that emphasis is placed upon the fact that the probationers receive in the home that "motherly moral training of character" which Miss Nightingale has always insisted upon as so essential an element in any sound system of nurse training. Dr. Sharkey, the examiner, reports favourably upon the lectures and work done, the good attendances of lady and nurse probationers, and the striking eagerness to learn. We rejoice to see that, unlike some reports, the Nightingale expresses gratitude to Dr. Ord, Mr. McKellar, and Dr. Sharkey for their valuable lectures and services, and to the ward sisters and nurses, as well as to the Matron and Superintendent, for the satisfactory manner in which their several duties have been performed. During 1892 90 probationer nurses have been attached to the school at St. Thomas's Hospital, of whom 18 resigned or were discharged as unsuitable, and 33 completed their year's training. Thirty-nine nurses, of whom 20 were special or lady, and 19 nurse probationers remained in the school at the end of 1892. Thirty-three probationer nurses were entered on the Register of Nurses after completing their training. It is satisfactory to note that a Register of Nurses is kept at St. Thomas's, as it ought to be kept at every well-administered hospital and nurse-training school all the world over.

### CARELESS CORRESPONDENTS.

There is a want of consideration about some correspondents, who make all kinds of inquiries, send long testimonials of the merits of their friends, and do other things appertaining to their own interests, without taking the trouble to enclose their name and address. We have so many anonymous correspondents of this class that we request our readers finally to understand that from this date all anonymous communications will be consigned direct to the waste paper basket.

### MISS VICTORIA JONES.

It will be a matter of regret and sympathy for many readers to hear that Miss Jones left her work at Guy's Hospital to take her sister abroad, in consequence of failing health. Miss Jones was connected with Guy's Hospital for thirteen years, and as the following resolution of the Governors has never been made public we have pleasure in abstracting it from the report of the treasurer just issued: "Resolved, that the Governors accept the resignation of Miss Jones of the office of Matron with much regret. They desire to record their sense of the zeal and activity shown by Miss Jones in every department under her charge during her ten years' tenure of office. Many useful reforms have been carried out at her suggestion, and through her personal influence the position of their nursing staff has materially improved and its members elevated to a higher sense of devotion to duty."

### MATERNITY NURSES.

For three years the patients at the Lying-In Department attached to Guy's Hospital have had the advantage of the services of two maternity nurses supported out of the earnings of the nurses upon the Guy's private nursing staff. These maternity nurses are sent out under the direction of the obstetric residents to visit the most neglected and deserving cases, and during 1892 the treasurer reports that no less than 250 of these cases were attended by them. The nurses made 3,300 visits altogether during the twelve months in question. In connection with this work at Guy's there is a Maternity Samaritan Fund, established by the present Matron, which helps to alleviate the state of destitution in which many poor women are discovered. Gifts of clothing, suitable for mothers or infants, and of money to defray the cost of necessary food and medical comforts will be gratefully received by the Lady Superintendent, Miss Swift, 14, St. Thomas Street, S.E. We could wish that all hospitals having a private nursing staff would speedily follow the excellent example thus set by Guy's. The work is essentially one with which women must sympathize, and we hope many readers may be moved to send Miss Swift some contribution in money or goods without delay.

### VILLAGE NURSES.

Lady Winchelsea and Miss Whitmore Jones, of Chastleton, have been engaged in an instructive correspondence on the work, position, and housing of village nurses. Lady Winchelsea, as we think rightly, favours the plan of the Jubilee Institute, whereby the nurse takes the most serious cases first in her daily rounds, and stays as long as she is required in confinement cases. The Queen's Nurse, with the aid of the donkey or pony cart, can undertake four or five villages within a radius of about four miles, carrying her food, of course, with her. On the other hand, Miss Jones is an advocate of the Ockley system, which provides that the nurse shall spend the whole of her time in the house of one patient at a time. The Ockley nurses (?) are paid 10s. per week, and the patient provides board, lodging, and conveyance. This latter system shows much to desire. It leaves out of account the many chronic cases, often the most suffering, deprives the nurse of her independence and "home of her own," and, in fact, is not a nursing system at all in any proper meaning of that term.

### "THE HOSPITAL" COMPETITION FOR NURSES' HOMES.

This competition is exciting a good deal of interest in the hospitals and schools, and those of our readers who desire to see the conditions, and to understand its objects, should refer to The Hospital for July 15th, page clvii., and for July 29th, page clxxvii. It is attracting much notice in various countries, and we should be glad to receive further correspondence or inquiries in regard to it on any point affecting the competition. The *British Medical Journal*, the *New York Medical Record*, and the *Trained Nurse* all give full details of this competition, which promises to be the most interesting and instructive effort yet made by trained nurses. Competitors must send their names and addresses, by November 1st next, to Dr. Billings, United States Army, Washington, U.S.A., plans and papers being sent in to Dr. Billings not later than January 1st, 1894.

### BRISTOL ROYAL INFIRMARY.

On Thursday, August 17th, the nurses of the Bristol Royal Infirmary were the guests of Mrs. Watson Williams, at Clifton, on the occasion of the distribution of prizes and certificates gained at the close of a course of lectures to nurses by Dr. Watson Williams. The Rev. Canon Ainger, who had kindly consented to give away the prizes, expressed his warm interest in the nursing profession. In the course of his remarks he assured the nurses of his sympathy with their work, and spoke to them of the great development that in the last fifty years had taken place in their profession, and how, by the work being taken out of the hands of coarse and ignorant women, and made a vocation worthy of engaging all the powers of the most cultivated and intelligent, a great responsibility and trust had been committed to them. They should remember that with many of the patients their stay in a hospital was possibly the first time in their lives that they had been brought under softening and refining influences, and it therefore lay in the nurse's power, not only to minister to the body, but also to influence them for good in many ways. The Canon further urged upon the nurses to use every means in their power to elevate their work, and to prevent them from falling into the monotony of a dull daily routine. Dr. Shaw afterwards returned thanks to the Canon and to the kind hostess who had provided the pleasant entertainment, which was thoroughly enjoyed by all present.

### AN AMBITIOUS COTTAGE HOSPITAL.

We have tidings of a very "go-ahead" cottage hospital in *The Trained Nurse.* The Newton Cottage Hospital, in the United States, has a training school for nurses. Here the first series of diplomas were given to successful candidates in June last. A large company was present on the occasion, and after the "graduating exercises," a poem was read, written by one of the nurses.

### A PROOF OF USEFULNESS.

We are interested and gratified to have frequent proof that our columns are really regarded as a source of information to nurses. We are glad that it is so, and wish our readers to know that we are pleased to afford all the help in our power. The latest example of reliance upon us comes in the shape of a request for information and advice respecting New York hospitals, the inquirer hailing from San Francisco.

### A HERO FROM A CONVALESCENT HOME.

We have just received from the Withernsea Convalescent Home, near Hull, the story of a gallant act by one of its inmates. It appears that two children whilst bathing with their aunt ventured out too far. The aunt, perceiving their danger, called for help. Her cry was responded to by one of the patients from the convalescent home who was on the beach. This was a man named Dove, who was recovering from a compound fracture of the ankle, and still disabled by it. In spite of his condition, he plunged into the water, and succeeded in rescuing one of the children, the other having disappeared from view. The conduct of Dove was much appreciated in the vicinity, and a collection of £3 2s. was made in his behalf, and most gratefully received, as he had been long out of work.

### NURSING AT DUNDEE.

The *Dundee Advertiser* publishes an illustrated account of the Dundee Royal Infirmary. This includes a sketch of the New Nurses' Home in the grounds of the infirmary. The ten nurses who now occupy the Home are indebted mainly to Mrs. Gilroy, of Castleroy, for their habitation. The furniture has been carefully chosen, and the dwelling is a comfortable one. The nurses in the Home form part of the private nursing staff at the hospital.

### SHORT ITEMS.

The lady pupils at Guy's are quite a source of profit to the hospital. Their fees last year added £1,031 to the income of the institution.—One of the newest of the nurse training schools of America is that at Cincinnati, under the superintendence of Miss Olive Fisher.—The nurses of the Victoria Nursing Institute at Taunton, after successful examination, received their prizes at the hands of Dr. Edward Liddon.—An open-air musical festival, in aid of the funds of the Heywood Sick Nursing Association, was held last week in the Queen's Park, Heywood, where there was an attendance of over 8,000 people. The Local Government Board have sanctioned the appointment of nuns as nurses in the Kinsale Union Infirmary, Ireland. A wish has found expression from nurses who have trained at the City of London Lying-in Hospital that a memorial shall be erected at Highgate Cemetery, as a mark of their love and gratitude to Miss Allen, the late Matron. Any contributions to this end will be gladly received by the Secretary, Mr. R. A. Outhwaite, at the hospital.—We regret to hear of the death, after a long and patiently-borne illness, of Mr. Nevill, of Hove, Brighton. He was a kind friend to nurses, and a constant subscriber to the Sick Nurses' Bed Fund, and will be much missed.

### Practical Information Column.

[We propose to answer questions addressed to us on matters of interest and importance to workers in the nursing field in this special column. Enquiries should be sent to The Editor, 428, Strand, W.C.]

### THE PARISH NURSE.

MR. C. S. CHAUNDLER writes from Hampshire as follows:—It is in contemplation to establish in this parish a certified nurse for the service of the poor, and I should be greatly obliged if some of your readers with experience in this matter would kindly give me information and advice either privately or in your columns. The funds are those of a public charity, and while economy is a point, efficiency is the first consideration. I should like to know in what quarter we might best apply, what salary would be expected, and what services might reasonably be required? Would it be practicable if a house were provided to have, say, two rooms furnished and acute cases nursed there? Is it customary to demand any fee in part payment from cottagers applying for the services of the nurse? The parish is chiefly agricultural and of considerable extent—population under 3,000.

### BY MRS. DACRE CRAVEN.

We sent Mr. Chaundler's inquiry to Mrs. Dacre Craven, who has very kindly written the following article in reply:—

District nursing, or the care of the sick poor in their own homes, is now generally recognised as being a necessary adjunct of the work of the dispensary or parish doctor, it being an almost universal rule in district nursing institutions that "no case is retained on the books which is not under a qualified medical practitioner." When the case has been sent by a medical man his orders are at once taken, and when not the nurse communicates with him and obtains his instructions in writing. These are the original rules of the Metropolitan and National Nursing Association to provide trained nurses for the sick poor in their own homes, and have always been rigidly followed by the nurses of this association since its foundation seventeen years ago.

These nurses "start on their rounds" at eight o'clock in the morning, and work eight hours a day. Upon entering a patient's room the nurse takes temperature, pulse, and respiration, and the usual "notes" (if she has been desired to do so) for the medical man in charge of the case. She then removes the linen worn during the night, that it may be well aired and freshened before being replaced. She sponges the patient between blankets, takes precautions against bed-sore, makes the bed, applies any "dressings" required, combs and arranges the patient's hair, and cleanses teeth and gums; then sweeps and dusts the room, ventilates it, and empties and washes all utensils, dirty glasses, &c. (and, when necessary, disinfects utensils and drains), sweeps up the fire-place—in some cases she has to make and light the fire—fetches fresh water, and fills the kettle. When necessary she prepares the beef-tea, light puddings, or drinks required. The time the nurse stays and the attention she gives to each patient depends on the nature of the particular case, as in some cases the relatives of a patient are taught to keep the room in nursing order, and how to ensure good ventilation, &c. In others, the patient may only require the services of a nurse to wash and dress some old wound or abscess, and and is able to keep herself and room clean and tidy.

A well known surgeon on the staff of the Middlesex Hospital for over twenty years, has described the class of cases in which district nursing meets a want that cannot be supplied in any other way, by bringing skilled nursing to patients suffering from consumption, spinal disease, or from prolonged joint disease, burns, scalds, old wounds, abscesses, paralysed and bed-ridden cases, chronic diseases which cannot be admitted into hospitals, and another and still more depressing class, because more hopeless, those suffering from cancer. When such patients have the daily skilled attention of a really well trained nurse they are not only spared much additional suffering, but their lives are brightened and cheered by her daily visits, visits always made about the same hour, so that they could be looked for and reckoned upon by the patient.

In district work the doctor rarely meets the nurse, except by special appointment, and his orders are usually given in writing. The district nurses must therefore be so well trained that no change can take place in any patient without being accurately noted and described by her, ready for his visit.

Various institutions throughout the country have been founded to train and supply district nurses, Liverpool being the birthplace of the movement, but the distinctive feature of the Metropolitan and National Nursing Association is that the nurses are gentlewomen, living together in "homes" under a trained superintendent, and that they never give "relief," only nursing service. That service is of the highest obtainable character. In other institutions the nurses are taken from the lower middle classes.

The average salary of a district nurse is £30 per annum, with board, lodging, washing, and uniform The cost of lodging differs in different localities, but "board" is computed at the rate of board wages for upper servants, and the cost of uniform averages £5 per annum.

In country parishes, where there is a scattered agricultural population of about 3,000 or 4,000 people, there is seldom work enough to occupy the full time of one nurse, unless she undertakes confinement cases, or there is an outbreak of fever. In the former, special limitations have to be placed by the medical man as to her attendance at other cases, from the risk of puerperal poisoning; and in the latter, if attending scarlet fever, or other infectious disease, she ought not to attend any other case until she has been properly disinfected and performed a modified quarantine.

In a few instances the combination of a cottage hospital and district nursing has been tried. But "No one can be in two places at once." If the patients in the hospital are "acute cases," needing skilled nursing night and day, they ought not to be left alone even for an hour. They would be better cared for in their own homes, especially if they could have hospital appliances lent to them, and the necessary skilled nursing twice a day or oftener. In country parishes it is better if three or more parishes combine, and employ at first one district nurse among them, adding another if it should be found necessary. If two or more nurses are employed in agricultural parishes, the long distances they have to walk, and the naturally trying and arduous nature of their work, unfit them for catering or cooking for themselves, and make them far less fit to wait upon and tend "home patients."

To preserve health and the even temper so necessary in nursing work, nurses must daily have real leisure, and change of thought and occupation. It is therefore a common rule that nurses are not to "talk shop," that is, discuss their patients, &c., when they meet for meals. Where it can be arranged it is best that the village nurse should board with some lady in the parish, or in the doctor's or clergyman's family, or in the cottage hospital, where there is one. The nurses in charge of the latter should follow the ordinary hospital rule, and have two hours daily for change and exercise, so that they may return to their wards refreshed and invigorated.

It is a common delusion among the working classes, as among their richer neighbours, that any woman can "nurse" when required; and it is, as a rule, not until they have felt the difference between skilled and unskilled nursing that they are willing to pay for such services, although willing to do so for "monthly nursing" in confinement cases. Among those who are fairly well off for their station in life, there are some who will gladly pay a small sum, the amount paid usually being determined by the medical man in charge of the case, and varying from sixpence to ten shillings weekly; but it has been found by experience that where small payments are made and received, larger donations and subscriptions fail.

The Rural Nursing Association (Miss Oldham, Secretary, 12, Buckingham Street, Strand) supplies district nurses of the ordinary class for country parishes only. The Metropolitan and National Nursing Association also supplies trained nurses for country as well as town parishes, but these nurses are of the higher educated class exclusively. (Address the Hon. Secretary of the Metropolitan and National Nursing Association, 23, Bloomsbury Square, W.C.) The Queen's Jubilee Nursing Institute, Regent's Park, supplies nurses of the same class as the Rural Nursing Association. In these, as in all well-organized district nursing institutes, the nurses work eight hours a day, have eight hours for sleep, and at least two for leisure, and they only work under qualified medical practitioners.

### The Benefits of Alumnæ Associations.

By Miss McIsaac, Illinois Training School, Chicago.

In presenting this short paper on the benefits of alumnæ associations, I must plead an ignorance of any further knowledge and experience than has been acquired by an eighteen months' connection with our own society. I do not wish to set it before you as an example, but rather for criticism and suggestion. Alumnæ societies have been started from several schools, but it is yet early to decide which plan is most successful. We are indebted to the New Haven School for many suggestions.

In our constitution we pledge ourselves, first, to "support by our personal efforts and interest, the organisation to be known as the Alumnæ Association of the Illinois Training School for Nurses." The first article announces our objects, which are:—

First: The union of graduates for mutual help and protection.

Second: To advance the standing and best interests of trained nurses, to co-operate in sustaining the rules of the directory, and to place the profession of nursing upon the highest plane attainable.

Third: To further the interests of the Illinois Training School by giving it our hearty support to make it the foremost among such institutions.

Fourth: To promote social intercourse and good fellowship among the graduates, to extend aid to those in trouble, and to establish a fund for the benefit of any sick among our members.

If the high sentiments expressed in this first article be carried out, how wide the field and how infinite the benefit! To quote further:

"Graduates of the Illinois Training School in good standing are eligible for membership, but any person upon the payment of 10 dollars may become an honorary member, and upon the payment of 50 dollars a beneficiary of the association, the amount thus obtained to be used toward the permanent endowment of a room in the Presbyterian Hospital. The initiation fee is 5.00 dollars, payable to the treasurer upon admission. The annual fee is 3.00 dollars, payable at the annual meeting. These fees to constitute the sick benefit fund. In case of necessity the executive committee can make an assessment to defray the burial expenses of any member."

In eighteen months we have paid out about 275.00 dollars, and have 519.70 dollars in our endowment fund, and 301 dollars in the sick benefit.

The officers to consist of a president, vice-president, secretary and assistant, and treasurer, elected by ballot at the annual meeting, and to hold office one year. The President to preside at all meetings, and the officers of the association to constitute the Executive Committee. A visiting committee consisting of one member from each class appointed by the President, to visit sick members, ascertain their needs and see that they are properly cared for. It shall be the duty of the Executive Committee to investigate charges brought against any member, and if they find such member guilty of conduct unbecoming a nurse, they shall present the facts to the society for action, but no member shall be recommended for expulsion until she has had notice and opportunity for a full hearing before the Executive Committee.

The amount allowed a member from the sick benefit fund to equal the cost of a private room at the Presbyterian Hospital, or if unable to enter the hospital, a sum not to exceed 10 dollars per week for a period of six weeks. In case of necessity the Executive Committee may increase the amount and extend the time. Applications for sick benefits to be made to the Secretary, and by her to the Executive Committee.

This is an outline of our purposes. It needs no argument to convince us that "in union lies strength," and "in knowledge power." We have for ourselves a most honourable calling, one which compels respect of all mankind. We are just beginning to realise how much higher we may go.

The system of training will soon be enlarged in every way. The successful nurse will be she who keeps abreast of the times. How better can it be done than by the maintenance of alumnæ associations?

The difficulties are our indifference and lack of enthusiasm. The routine life of a nurse tends to narrowness, and that to selfishness. The irregular habits interfere with study and recreation, and it requires enthusiasm and a realising sense of the immense improvement to be derived from concentrated efforts to lift us out of a meagre intelligence and commercial spirit. If every school establishes alumnae associations the obstacles to a national organisation will rapidly disappear, and if only members in good standing, active members, not merely those who pay their fees but never come near the meetings, are eligible for membership in the National League, each will be a stimulus to the other. Then let the national organisation establish a code of ethics which shall govern all auxiliary bodies. It can define the conditions constituting "good and regular" standing in the auxiliary associations, thus establishing a high grade of excellence for membership; it can provide rules and procedures for discipline. The practical working of a National League will bring to the front the best material for the heads of schools.

We have the example of the honourable bodies of the medical profession. Our societies should be based upon simple business principles, but let us guard against the idea of their being only a medium for financial aid, and still further should we avoid any idea savoring of charity in the ordering of sick benefit funds. It is mutual help, not charity.

The furtherance of post graduate study should be encouraged in every way. During the winter our society had the benefit of several lectures from eminent medical men, notably upon cholera and yellow fever epidemics, other contagious diseases and the nursing of abdominal surgery; these were given most cheerfully by busy doctors and were much appreciated. We need better literature and more of it on nursing. If we demanded it by our better intelligence it would be forthcoming. The really good books on nursing could be counted on one's fingers. During the past six months two volumes by nurses and for nurses have emanated from the Johns Hopkins School, which throw strong light upon dark places; we needed them. Let us show our appreciation. We have but one journal which all schools should feel it a duty to support in every possible way. The heads of all schools should bring these associations to the notice of undergraduates long before they leave the school.

We have fought for firm footing. We can by united effort lift ourselves to honourable permanency. "United we stand, divided we fall."

### The World of Nurses.

As seen by an Unprofessional Correspondent.

### III.—NURSES AND DRESS.

What is the function of dress? Surely that it should be above all things suitable clothing—suitable to our position and our work. A long train is elegant, and not out of place in a drawing-room where the owner has little or no-thing to do; but it is very unsuitable in dirty streets, and no lady other than a nurse would trail her skirts along a dirty pavement or a dusty floor. Yet one almost invariably sees nurses in long dresses. But to make matters clear, let us inspect the nurse's uniform from head to foot.

Indoors, caps vary from imitations of various "sisterly" and nun-like headgear to the former housemaid arrangement of four inches or so of net and lace. Gowns are of stuff or print, more or less suitable as regards material, but nearly always long enough to hide the feet, or sweep up dust and germs. Possibly the modern nurse has not the courage to show her feet when shod comfortably, although I have observed that often the gown is short in front, displaying ornamental shoes with high heels. The sister of charity wears ugly, comfortable, neat, and clumsy shoes, and so does not suffer such agonies from standing. Let that pass and consider once more the gowns. Is white *piqué* suitable for London? Dresses thereof cost double to start with; and to be clean two a week are needful, thus doubling the washing, which is hard upon the nurse, or extravagant as regard the hospital, whichever happens to pay the laundress. Also imagine for one moment the absurdity of a white gown trundling about in this smutty city, and in the loathsome underground railway and dirty omnibuses! Then, again, consider the noise which those sheaves of pencils, keys, scissors, &c., make jingling at the side. Often have I sat by the side of "sisters" in foreign churches, sisters of charity with rosaries added to the scissors, &c., and never have I heard this disturbing jingling; and why? Possibly because the tools hang upon ribbons instead of steel chains.

As to the outdoor uniform, it is arranged, apparently, not as a protection to the wearer, but as an attraction. Formerly the "poke" bonnet, or the more modern neat close straw bonnet, was put on over the cap to save time, hence the white strings of the latter showed, and a veil was put over the face when necessary. Now white strings are sewn on to the bonnets for show, and a long strip of gauze of various colours hangs down the back for effect—both arrangements being the most consummate shams and pretences; the one, strings of a cap which does not exist; the other a veil which could not be placed over the face. This is what we have come to, and one need only turn over the pages of certain advertisements to see the fascinating costumes worn by the modern nurse—if she always be a nurse!

Then the cloak; think of its varieties, some even having long hanging sleeves of no use whatever but to blow about. By the way, that is another element of the uniform cloak, that it should not be attached anywhere, and so prevent the wind displaying the aprons underneath. Supposing we worldlings wear aprons at home to protect our dresses, we take them off when we go out; we know that our street garments are not too clean; but the nurse takes care to put on an exquisitely white apron and cuffs for her walk. We worldlings again, when we go to the gallery of a theatre, dress ourselves in our least noticeable hats and bonnets; the nurses sit up in all the glory of white strings, cuffs, collars, jinglings, and *et ceteras*, and one has been known to resent, forsooth, that she was not admitted into a box at the opera in her uniform! Why should she? Is uniform "evening dress"? Why should not a lady artist as well present herself in her painting blouse, or a man in his grey suit? Uniform is essentially a working costume, and it is, therefore, a gross piece of affectation, and shows a most improper desire to be conspicuous when a nurse wears it in public while the rest of the world is clad in the conventional evening dress. As a nurse, a woman is the observed of all observers. Dressed in ever so humble or simple a gown of conventional form, she passes in the crowd—probably the last thing she wishes to do—just as people become members of small dissecting religious societies, rather than remain in a large church of which they are insignificant members, and lost to view. The humble Churchman is content to be a small particle of a large body, and the right-thinking "sister" is content to be a useful atom, a working bee in a great hive unnoticed, except as a worker, and ready to sink into a mere memory when the time comes for her place to be filled up by another. Marriage to this woman is a secondary matter; if it comes as a happy accident, well and good; it is the happiest condition. But she does not seek it. She does not court it by the curling of hair or the wearing of becoming costume, and it is the last thing she thinks of when she enters a hospital. No one can say that the old St. John's outdoor uniform was attractive, yet it did not prevent some of the sisters marrying. There is no reason why they should not; but there are many reasons why they should not flirt with students and junior medical officers, in corridors, on staircases, and by the bedsides of patients. The whole gist of the matter is the abolition of outdoor uniform. The usefulness of it in a public place in cases of accident is small as compared to the lowering of the tone of the profession. If it be of any advantage to the public that nurses should be able to declare themselves nurses in case of need, let them wear some badge round their necks underneath their cloaks. Matrons complain of the numberless women who apply to them, and who even enter hospitals, without the slightest eligibility for the work; let outdoor uniform be abolished, and the ranks of the candidates will be immediately thinned of the very women it is desirable to keep out of the profession. To be lost in the crowd in the streets, to be obliged to dress like the rest of the world, and pass by unnoticed would soon clear the ranks of the offensive husband-hunters and the partially and indifferently-trained nurse. Why is the nursing profession so popular just now and domestic service so much the reverse? Surely not the superior wages of the nurse, nor the amount of freedom, nor the shorter hours. Very few servants work twelve hours a day. Vastly few go from week's end to week's end with only a couple of hours off now and then. It is because the nurse's is a more genteel calling; she is a lady, and who can say she is not when she belongs to a private nursing agency? It is because she is under no control in a private house, and can be waited upon by the servants. Ask the high-class domestic servant what she thinks of the nurse, and she will tell you she is often an "upstart."

There is another and very serious reason for the extinction of an outdoor uniform; it is a danger both to patients and public. No one knows who she may sit next in a public conveyance or at a theatre, and if a nurse goes out in her hospital uniform she may carry infection to the healthy or to the sick—both considerable risks.

Nurses belong to a calling which professedly is noble, and they should set their worldly sisters an example in modesty, as they so often do in self-sacrifice and unselfish devotion to their weaker brethren.

### "The Truth about the London Hospital." —*Pall Mall Gazette.*

"The charges against the London Hospital are in reality but the attempt of one woman to get her knife into another."

Sir Edmund Hay Currie.

Wherever worth and merit shines,
There haggard malice hatches base designs;
Spouts forth her venom, to o'erwhelm with shame
The rising brightness of a noble name;
Sullies with envious mud the robe of white,
And darts her slanders at the child of light.

The *Pall Mall Gazette* has shown commendable fairness by throwing its columns open to both sides. It is a little amusing, however, that the *Pall Mall Gazette* should have suppressed a statement as to its Special Commissioner's article published on the 29th ult., to the effect that "her spelling proves her nationality and so fixes her identity."

### Everybody's Opinion.

[Correspondence on all subjects is invited, but we cannot in any way be responsible for the opinions expressed by our correspondents. No communications can be entertained if the name and address of the correspondent is not given, or unless one side of the paper only be written on.]

### NURSES' UNIFORMS.

"A Wearer of the Red Uniform" writes: Much has been said of late respecting nurses' costumes. Outdoor uniform is not only a save but a great comfort to both hospital and private nurses. When nurse has perhaps only one hour for her constitutional walk, part of that time would be spent in changing her garments, whereas uniform enables a nurse to get more fresh air, and on returning she has only to slip off her cloak and bonnet and return to the sick room ready for duty. No conscientious nurse would think of going out with any garment that has been in contact with infection. Last week's correspondent, that signs herself "Aiphos," seems to think it is only worn to attract attention, and that no present-day nurse's life ought to be that of a nun's; she must not laugh, or when in omnibuses raise her voice so as to be heard by those sitting beside her; she must not, when in Regent Street or near the Burlington Arcade, be allowed to look in shop windows, and to think of going to a place of amusement would be far too great a crime. Outdoor exercise is not only beneficial to the nurse but to her patients. Occasionally there is no harm in going to a theatre; it enables her to have something fresh to talk about. As nurses we like to be bright and cheerful; when out we leave our nursing duties in the sick room or ward, which ever may be the case, not forgetting on our return to devote our whole time to the care and comfort of our patients.

### A HOME FOR INCURABLES.

"Miss E. Petrie" writes: While thanking you very much for your notice of our little Home in last week's issue of The Hospital, I shall feel greatly obliged if you could spare a line or two to explain that we receive only "the destitute and the dying." After more than twelve months' unsuccessful hunting for a house which should have a few green trees, and a fair amount of quietness about it, and yet be in a central position, the first part of that ideal has been regretfully abandoned. We have taken this house, 50, Osnaburgh Street, close to the Portland Road Station, which has, after some alteration and addition, been formally opened under the above name, and which is large enough easily to provide for 15 patients' beds, 7 for men and 7 for women, with 1 isolation room for any case of either sex for whom such an arrangement may be desirable. It will be absolutely free, the only pecuniary condition being that the friends must provide for removal and interment after death. It is not intended for chronic incurable diseases, such as paralysis, &c., but for men and women who cannot reasonably expect to live more than a few weeks or months, who shrink justifiably from the Workhouse Infirmary, to whom no General Hospital could spare a bed, and who are too poverty-stricken to procure for themselves the medical attendance, nourishment, and nursing which might, in some measure, alleviate the pain and weakness of their last days. All cases fulfilling these conditions will have to be seen by one of the visiting doctors before admission—the final decision resting entirely in Mr. Barrett's hands. As it is hoped that people of every Church, sect, and creed may find shelter in St. Luke's House, it is an understood thing that whatever form of religious help and teaching they prefer shall be obtained for them. The West London Mission, from whose medical department this institution sprang, will undertake a short weekly service, also the visiting of patients at stated times for their spiritual instruction and comfort. It is hoped that some of the neighbouring clergymen and ministers will be willing to assist in this way at other times. We have not dared, knowing that in this hospital there will be no "convalescents"—to estimate the cost per bed at less than £50 a year, £750 for the fifteen beds with which it is proposed to begin. Towards this we have no endowments, but depend altogether on our own friends and the general public. The inmates will always come from a very widespread area, limited indeed only by their feeble powers of journeying, and we earnestly hope to find subscribers in still more varied places and positions. The alterations and repairs, and the purchase of such furniture as must be of the "best hospital make," will exhaust a large proportion of the fund now in Mr. Barrett's hands; and though numerous and valuable gifts have already been sent towards outfitting, we shall still be grateful recipients of all articles, decorative or useful, suited for ward or nurses' use, and of any amount of linen, &c., new or old, for the beds or the poor people who will occupy them. It has been calculated that it will cost almost exactly £10 to "fit up" one bed, *i.e.*, bedstead, bedding, new blankets, quilts, sheets, and pillow-cases to change, waterproof sheeting and water pillow, bed-rest and bed table; and, of course, anyone who gives or gathers £50, will keep one bed "going" for one year. The house will be ready for the reception of patients in a few days' time, *i.e.*, whenever the workmen have finished, and the nursing staff complete. Forms of application and lists of rules will then be supplied to anyone writing to me here for them. We shall be glad to show the hospital any afternoon after half-past two p.m.

### Novelties for Nurses.

### WOOLLEN UNDERCLOTHING.

Messrs. Fleming Reid and Co., The Worsted Mills, Greenock, N.B.

Messrs. Fleming Reid have made our task of noticing easy, as it would be impossible to speak in any terms but those of the highest praise of their most admirable manufactures. We can unhesitatingly say we have met with nothing to beat them, either in quality or price, and we cannot advise our readers of both sexes better than to send for a catalogue of Messrs. Fleming Reid's specialities. The firm supplies every possible form of woollen underclothing; beautifully finished elastic and soft vests for ladies, made of the best wool we have seen for the purpose, are to be had for only two shillings. Combinations, which of equal warmth and softness we have not been able to procure ready made for less than eleven shillings, are here sold at five shillings, and these possess the added advantage of being far better shaped than any we have yet met with. Drawers are equally nice, and so are petticoat bodices, made, high or low, of the finest elastic stockingette. Body-belts, which for Anglo-Indians are a positive necessity, and which are now largely adopted by others, are rendered far more convenient than flannel belts, as they permit of fit without restraint, and moreover, as they allow ventilation, are certainly more hygienic. These also only cost the small sum of two shillings. Lastly, we would ask our readers to make a trial for themselves of some of the produce of the Worsted Mills before they judge us too enthusiastic in our recommendation, believing, as we do, that they will share our pleasure in knowing where to go for underclothing of such excellence and moderate price.

### "THE LEICESTER" ACCOUCHEMENT BINDER.

This binder (A. De St. Dalmas and Co., Leicester), made after the suggestion of Dr. Cory, of Bournemouth, is a very great improvement on those generally used, in fact, it is all that could be desired. It is antiseptic, light, soft, firm, durable, and inexpensive, which qualities should recommend it for universal use. Any chemist will supply it.

### Death in Our Ranks.

The House Governor of the General Hospital, Birmingham, writes: We have to record the death, on the 20th ult., of Miss Elizabeth Armstrong, aged 37, staff nurse of the General Hospital, Birmingham, after an illness of six weeks. Nurse Armstrong was trained at Newcastle Infirmary (three years), and then served for fourteen months at the Abroath Infirmary. She was appointed a staff nurse at the General Hospital, Birmingham, on February 11th last, and died there, to the great regret of the authorities and officers of the hospital, and of her numerous friends amongst the nursing staff. A short service, conducted by the chaplain, was held on Tuesday evening in the hospital chapel, at which, in addition to her two sisters and three intimate friends, two of the physicians, the House Governor, the residents, the Matron, and all the nurses who could leave their wards were present. The coffin was covered by a handsome cross of white flowers from the Matron and nurses, and surrounded by wreaths from the House Governor, the residents, and a former nurse. The body was removed to Edinburgh on Wednesday morning. At the next meeting of the House Committee they passed the following resolution: "That the House Committee have heard with regret of the death of Nurse Armstrong—an efficient and valued staff nurse—and instruct the House Governor to convey their sympathy and condolence with Mr. Armstrong and the members of his family in their bereavement." Her late Matron, when Nurse Armstrong applied at the Birmingham General Hospital, wrote: "In these days of dissatisfied nurses you may be glad to hear that Nurse Armstrong's strong point is her thorough contentment with her surroundings. She is not one of those people who expect to have everything to their mind. Besides being a good nurse, she has shown considerable tact in the management of the patients and their visitors."

The recently opened Cottage Hospital at Malmesbury has sustained a severe loss in the death of the Matron, Mrs. Herbert. She had greatly endeared herself to the inhabitants of Malmesbury, among whom she had nursed for some time, while awaiting her appointment as Matron. Her death, which was caused by peritonitis, took place on Sunday morning, the 20th ult.

### Minor Appointments.

Central London Throat Hospital.—Miss Helen E. Court, late Sister at the Leicester Infirmary, has been appointed to the Matronship of the Central London Throat Hospital.

Zanzibar Hospital.—Miss Margaret Breay has been appointed Matron of this hospital. Miss Breay was trained at St. Bartholomew's Hospital, and has since held the post of Acting Matron at the Metropolitan Hospital, Kingsland Road. Miss Breay holds the diploma of the London Obstetric Society.

Huddersfield Infirmary.—Miss Edith Thomas has been appointed charge-nurse of the male medical ward, with charge of the operation room. Miss Thomas was trained at Durham, spent a year and nine months as charge-nurse of the female surgical ward at the Bradford Infirmary, and comes to Huddersfield from the Royal Infirmary, Edinburgh, where she has gained additional experience as assistant nurse.

Watford Cottage Hospital.—Miss Emily Atthill was appointed Matron of this institution on August 20th. Miss Atthill received her training at the Macclesfield General Infirmary, and at the Adelaide Hospital, Dublin. More recently she has worked at the Royal Derbyshire Nurses' Training Institution. We wish Miss Atthill every success in her new work at Watford.

Miss Cheatte asks us to say that although she has been appointed Lady Superintendent of the Trained Nurses' Association at Clapham, the post of Principal is still held by Mrs. Chapman, who is also the owner of the home.

### For Reading to the Sick.

### PRAYER.

To the true Christian prayer is the greatest solace and comfort in sickness, and those who cannot, and those also who do not care to lay claim to the title, would be right glad if they could share in the consolation which intercourse with God affords. But from carelessness and neglect in the past the habit of prayer first learnt at the mother's knee has not been kept up, and so while the sufferers are wondering *how* they shall ask, and *what* they shall ask for, the time has flown and the opportunity is lost. It is well for everybody to have some set form in their minds, so that they can fly at once to the throne of grace and pour out their sorrows when the desire arises in the heart.

Our Lord's disciples must have felt a like inability to put their thoughts and wishes into words, as they asked Him, "Lord teach us to pray," and the answer came in that perfect example, which by them and by us in the present day is called "The Lord's Prayer." It is indeed perfect both in itself and as a pattern on which every other petition may be moulded, for it covers all our wants, and has the fine characteristics of prayer in that it is safe, suitable, orderly, devout, and humble. As St. Cyprian says, it is "Like the prayer of a friend to a friend, familiar and yet respectful, asking of the Lord that which it is especially His to give, so that we can never return from making use of this prayer without deriving some benefit from it."

We learn then that our prayers should be suitable to our real needs, for it not unfrequently happens that God does not grant our petitions because He knows that the things which we ask for are not desirable for us. "Ye ask and ye receive not because ye ask amiss," says St. James, and it is not easy to know for certain what we may ask for, because we cannot be sure what is really good for us. But Christ has taught us once for all what we ought to ask for, and we shall find that whatever is fit for our happiness, and lawful for us to have, is contained in the Lord's Prayer.

When we think over its several petitions we are struck by their becoming order and arrangement. Christ had told men to "Seek first the kingdom of God and His righteousness"; therefore He teaches us to plead for heavenly things before we ask for earthly benefits. The form He puts into our mouths is so short that, if we are truly in earnest and have a fervent intention in our hearts, we shall not lose that devotion of mind and body without which our prayers are not acceptable to God. Next week we will notice the condescension with which we are treated by our Heavenly Father.

### Notes and Queries.

### Queries.

(182) *Medical Nursing.*—Will anyone kindly tell me of any hospital where I need not learn both medical and surgical nursing, but only the former?—*Daisy.*

(183) Can you tell me where I can get a book on infant feeding?—*Monthly Nurse.*

(184) *Daily Nurses.*—Is there a scheme for providing daily nurses for people of limited means?—*Visiting Nurse.*

(185) *Midwifery.*—What books should a nurse who has had training in a Maternity Hospital study in preparation for passing the L.O.S. examination?—*Dublin Nurse.*

### Answesr.

(182) (*Daisy*) *Medical Nursing.*—We think you had better not attempt what you propose, but if you do, apply to Miss Cook, Matron of the Seaman's Hospital, Greenwich.

(183) (*Monthly Nurse*).—Dr. Cheadle's "Artificial Feeding of Children" (Smith, Elder, and Co.) is an excellent book on this subject. "Hygiene of the Nursery," by Dr. Louis Starr (published by H. K. Lewis, 136, Gower Street), also contains good information. You do not comply with our rules.

(184) *Daily Nurses* (*Visiting Nurse*).—You had better apply to Miss Philippa Hicks, Nurses' Co-operation, 8, New Cavendish Street, Portland Place, W., who will give you the information you require.

(185) *Midwifery* (*Dublin Nurse*).—You would get valuable advice by writing to Hon. Sec. of Trained Nurses' Club, 12, Buckingham Street, Strand. Classes are held for the purpose of preparation. The Secretary of L.O.S., Hanover Square, would also give you information. You do not tell us what books you have already studied.

### The Muses' Looking Glass.

### THE LESSON LEARNT.

The *Idler* for August contains an interesting chapter of autobiography by Morley Roberts, author of the "Western Avernus," whose career seems to have been as picturesque as his person, if we are to judge by the portraits of him in his various rôles. Mr. Roberts desired to outrival Rossetti as a poet, but not the kindest friend would tolerate his verses. He had to earn his living less romantically till his health failed. "On February 27th, 1884," he writes, "I was working in a Government office as a writer; on March 27th I was sheep herding in Scurry County, North-West Texas." Four years later he returned to England no richer than he left it, though "I had tried some forty different callings, including 'sailorising,' saw-mill work, bullock driving, tramping, and the selling of books in San Francisco." Then he tried to play the "Ancient Mariner," with George Gissing for the "Wedding Guest," but with less success than Coleridge's original. Mr. Gissing, with laudable presence of mind, refused to hear the impassioned narrative, but bid him sternly "Go and write it down." Only on Sunday was he allowed to read aloud his week's work. In four weeks he completed the "Western Avernus," which is a true tale told red-hot by one still smarting from having been the hero of it. The book was accepted by Messrs. Smith and Elder, and published by them. And now, in the *Idler*, six years after, the author gives us the moral that he has since learnt from his own book. Which is that what then seemed his bitterest misfortune has proved the great boon of his life. "While I was gaining the experience that went solid and crystallised into the 'Western Avernus,' I was discovering much that had never been discovered before, not in a geographical sense, but in myself. Each new task teaches us something new and something more than the mere way to do it. To drive horses, milk a cow, make bread, or kill a sheep, sets us level with facts, and teaches us some reality. We are called on to be real, and not the shadow of others." "For that wild life, far away from the ancient set and bonds of social law which crush a man and make him just like his fellows, or so nearly like that only intimacy can discover individual differences, had allowed me to grow in a different way and become more myself—more independent, better able to fight and to endure. This is the use of going abroad and to places that are not civilised—they allow a man to revert and be himself. We live in a crowd here, and a man must be a rebel if he would be himself, and in the struggle for freedom he is likely to go under. In America there is a sharp contrast between city life and the life of mountain and plain. It is seen more clearly than in England, which is all more or less city. There are no clear stellar interspaces in our life here. But out yonder a long day's ride across the high barren cactus plains of Arizona teaches us as much as a clear and open depth in the sky. Of a sudden we run into a big town and staves are made gods for our worship. It is difficult to be oneself when all others refuse to be themselves. This was for me the lesson of the West. When I wrote the book I did not know it; but I see now what I learnt in that hard and bitter school, for I acknowledge that the experience was at times bitterly painful. It is not pleasant to toil sixteen hours a day; it is not good to starve overmuch; it is not well to feel bitter for long months. And yet it is well and good and pleasant in the end to learn realities and live without lies. It is better to be a truthful animal than a civilised man as things go. I learnt much from horses and cattle and sheep; the very prairie dogs taught me. They didn't attempt to live upon words and the ashes of dead things. They were themselves, and no one else, and were not diseased with theories or a morbid altruism that is based on dependence. . . . This is the lesson I learnt from my own book. I only went to America because the life of London made me ill. . . . Most of us hold to other men's theories instead of making our own. When Mill said, 'Solitude, in the sense of being frequently alone, is necessary to the formation of any depth of character,' he spoke almost absolute truth. Here we can never be alone, the very air is full of the dead breath of others. . . . Few who have read the 'Western Avernus' have seen what I think I see in it now. It has taken me six years to understand it and what it meant. That is the way in life—we do not learn at once what we are taught, we do not always understand all we say, even when speaking earnestly. In those hard days by the camp fire, on the trail, on the prairie with sheep and cattle, I did not understand how they called up in one the ancient underlying experience of the race, and like a deep plough, brought to the surface the lowest soil which should hereafter be a little fertile. . . . When I climbed with bleeding feet the steep slopes of the Western hills, my thoughts were set in a narrow circle of dark misery. I could not think of those who had striven like me, in distant ages. But the songs by the camp fire, the leap of the flame, and the crackling wood, the lofty snow-clad hills, and the long dry plains, the wild beasts, the venomous serpents, and the need of food brought me back to nature, taught me, as they teach us all, that the real and deeper life is everywhere, even in a city, if we will but look for it with unsealed eyes and minds set free from the tedious trivialities of this modern life." We can't all go to Texas, but we may all learn the lessons that "Cowboy Roberts" learnt there. Let us strive to be true, true to nature, he says. True to God's soul, that He has put within us, and will claim from us. Let us not merely imitate others, trying to be respectable because others are, but because we desire to train ourselves to do and be the very, very best that in us lies.

### Australian Notes.

(From Our Special Correspondent.)

The sharp financial crisis through which Victoria, and more especially its capital, Melbourne, is passing, has badly affected the contributions to the hospitals, and special appeals have been made to the public on behalf of the Women's Hospital and the Children's Hospital. Responses have been received from the whole colony, with the result that in a few weeks upwards of £1,000 has been subscribed for the first-named, and nearly that sum for the second institution. All Australian public charitable institutions are awarded a yearly grant from the Government equaling the amount of voluntary contributions and subscriptions received in each particular year, so that when these fall off the grant is similarly reduced and the institutions suffer doubly. By the special efforts mentioned the Women's and Children's Hospitals will have a good claim from the Government. On this occasion the Press is advocating the claims of the School for the Blind and the Institute for the Deaf and Dumb, both of which are suffering from the bad times. A very medical marriage took place on Tuesday, July 4th, when Dr. Emma Constance Stone, Melbourne, was married to Dr. Egryn Jones, Melbourne. Miss Stone received her medical education at Trinity College, Ontario, America, and studied for a time in London, where she passed the Apothecaries' Examination. On returning to her native country she commenced practice in Melbourne, and now has an extensive *clientelle*, principally, but not exclusively, amongst her own sex. Mrs. Jones intends to pursue the practice of her profession.

### The Book World for Women and Nurses.

[We invite Correspondence, Criticism, Enquiries, and Notes on Books likely to interest Women and Nurses. Address, Editor, The Hospital (Nurses' Book World), 428, Strand, W.C.]

The Lord of Humanity; or The Testimony of Human Consciousness; with Supplement on the Mystery of Suffering. By Frederick James Gant, F.R.C.S., Consulting Surgeon to the Royal Free Hospital. Second Edition. (London: Longmans, Green, and Co.)

There is an old epigram—Sir Thomas Brown alluded to it as "the common scandal of my profession"—which says that where there are three doctors there are two atheists. Undeniably it often seems that much dealing with physical suffering blunts the imagination, and that the doctor sees in his patient a damaged machine, which differs from other machines only in being conscious. With anything behind the immediate cause of the disease he has nothing to do, and even where he admits that a mental alteration may result from physical disease, he does not trace it beyond its presumed origin in some disturbance of nervous centres. But besides these men, among whom we will find our so-called atheists—our modern term agnostic is more true; they do not deny any possible cause, they simply affirm their ignorance of anything beyond their own experience—there is the third, to whom disease has interests beyond those arising from the action of drugs or the operations of the scalpel. It is to these that Mr. Gant belongs. A long life spent face to face with suffering has roused him to inquire into the deeper meanings of pain, and its possible spiritual effects. Sometimes his explanations tend rather to mysticism, as is, indeed, almost inevitable in dealing with a subject which at first sight seems obviously to give the lie to our conceptions of a good and merciful God. There can be no doubt that many tender souls are driven to scepticism or blank denial through this feeling — all for the want of a little knowledge of physiology, which sees primarily in all disease the violation of law. This, of course, carries us only one step further back, as Mr. Gant, with the imagination of a physician and a Christian, has perceived. The physical law is broken as a result of the moral law having been broken first; rules for conduct in the first place are rules for health in the second. But the law-breaking is not always direct; it may be vicarious. It is not the sufferer himself who has sinned, but some of his ancestors. This brings before us the whole problem of undeserved suffering, the inmost mystery of Christianity. To say that Mr. Gant has solved this mystery would be untrue. He has not succeeded any more than the theologians on the one hand or the saints and seers on the other; but, like the latter, he has found many lessons of consolation and encouragement in the contemplation of the Great Sacrifice. The attempt to realise the Divine consciousness in the presence of suffering and surrounded by sin is both reverent and sympathetic; the Divine side of the Saviour is not degraded by his humanity, nor is the human chilled by the presence of his Divinity. This book is not one of those dogmatic manuals in which the whole scheme of salvation is formally set down; it is rather a work of suggestion, reflection, aspiration. Written from the point of view of one whose life has been spent in studying and healing disease, it is peculiarly adapted for the sick-room, where we have little doubt that the thoughts suggested by it would be found full of help and consolation. As a

**THE BOOK WORLD FOR WOMEN AND NURSES**—*continued.*

testimony that there still live, in spite of modern accusations of coldness and scientific indifference, physicians who see the man behind the patient and the soul within the man, it has a value in no degree less important; and we recognise in it that special usefulness which the thoughts of a layman always possess in the public eye, as being more assuredly and incontestably genuine than many people will believe the most earnest utterances of the professed clergyman. We commend this book to all who are brought into contact with the sick.

A Handbook of Obstetric and Gynæcological Nursing. By the late Fleetwood Churchill, M.D. Revised and greatly enlarged and edited by Thomas More Madden, M.D., F.R.C.S.E. Pp. 216. Illustrated. (Dublin: Fannin and Co. Fifth edition, 1893.)

Dr. Churchill's work on Obstetrical Nursing, under the more familiar name of "Manual for Midwives and Monthly Nurses," is well-known to the nursing profession. It has been for some time out of print, and Dr. More Madden has undertaken the editing of a new edition. As was inevitable, Dr. Madden has found that much of the text as left by the author had to be rewritten and brought up to date, more especially those sections dealing with gynæcological operations, in regard to which the Listerian method has wrought such great changes. This has been done with great care and the new matter has been grafted on to the old in such a skilful manner that no irregularities and but few repetitions are to be detected. The instruction given is most terse and practical, and the nurse is not only told what to do, but also—often a matter of much greater importance—what *not to do.* A considerable amount of space is devoted to the important subject of puerperal septicæmia, and minute instructions given as to how this deadly affection is to be avoided. In the last fifty pages, the modern methods of gynæcological nursing are fully discussed and brought up to date. But a book in its fifth edition has passed beyond the scope of criticism, and we can confidently recommend this revised work to all interested in the subject of which it treats.

Sunshine and Shadow. By A. F. Hills. (Vegetarian Publishing Office.)

We think even some vegetarians may feel after perusing this work that it would be desirable to keep their vegetables out of literature. The rather diffuse history of the athletic young gentleman who at successive stages renounced the unholy joys of alcohol, fox hunting, pheasant shooting, meat and tea, is not calculated to convince any but those already pledged to the "great renunciation." Much eating of grain and such like produce appears to develop a desire to enforce a similar diet upon all one's friends. The story will therefore serve a laudable purpose as a gift-book in the hands of those who live to do credit to the principles it maintains.

Helps to the Study of the Bible. (Henry Frowde, Oxford University Press. 4s. 6d.)

It would be difficult to find a volume of equal size containing so much valuable matter as is enclosed in the small compass of these "Helps." It is in fact an entire library of Biblical knowledge, containing a wealth of information, only formerly obtainable in separate and costly works. To all who are engaged in giving religious instruction, the volume may be said to be indispensable, and to the large company of solitary Bible students it will prove an inestimable boon. Nothing could be clearer than the form of arrangement adopted, or the explanations, summaries, and multiform information supplied. Indeed, from the care displayed in this particular, the book is within the range of the humblest student, while representing the highest point to which modern science has attained in Biblical research. The plates, reproducing in facsimile authentic documents, monuments, pictures and portraits, illustrating the history of the Old and New Testaments, are specially valuable and interesting, and form a distinctive feature of the present revised and enlarged edition.

